# Comprehensive Analysis of Enhancer RNAs Identifies LINC00689 and ELFN1-AS1 as Novel Prognostic Biomarkers in Uveal Melanoma

**DOI:** 10.1155/2022/5994800

**Published:** 2022-02-23

**Authors:** Su Zhao, Hao Jiang, Jing Liu, Dao-yuan Li, Bing Li, Qiu-rong Long, Lei Zheng, Hao Gu

**Affiliations:** ^1^Department of Ophthalmology, The Affiliated Hospital of Guizhou Medical University, Guiyang, Guizhou, China; ^2^School of Clinical Medicine, The Guizhou Medical University, Guiyang, Guizhou, China; ^3^Shenzhen Eye Hospital, Shenzhen, 5180403 Guangdong, China

## Abstract

Enhancer RNAs (eRNAs) have emerged as key players in the pathology of several tumors, including uveal melanoma. Here, we aimed to explore the prognostic values of eRNAs in uveal melanoma (UVM) patients. The expressing data and survival data of UVM patients were downloaded from TCGA and GSE22138 datasets. The Kaplan-Meier methods with the log-rank test were applied to screen survival-related eRNAs in UVM. GEPIA was applied to analyze the associations between expressions of eRNA and disease-free survival. KEGG assays were applied to explore the potential signaling pathways of the key eRNA. The prognostic values of eRNAs were further explored by multivariate assays by the R package survival. The eRNAs were validated in pan-cancer. In this study, we identified 89 survival-related eRNAs in UVM based on TCGA datasets. Based on GSE22138 datasets, we found 27 survival-related eRNAs in UVM. Only two eRNAs (LINC00689 and ELFN1-AS1) were overlapped in both two datasets. The results of multivariate analysis revealed that both LINC00689 and ELFN1-AS1 were independent prognostic factors in UVM patients. The pan-cancer validation results further confirmed the prognostic values of LINC00689 and ELFN1-AS1 in eight tumors. Overall, we identified two novel UVM-related eRNAs, LINC00689 and ELFN1-AS1 which may serve as prognostic and diagnostic biomarkers of UVM patients for clinical decision-making.

## 1. Introduction

Uveal melanoma (UVM) is a malignant tumor that originates in melanocytes of the choroid plexus, ciliary body, and iris of the eye [1]. In recent years, its incidence displays an increasing trend with the dramatic shift of dietary structure and life style throughout the world [2]. To date, no effective treatments are available for metastatic cases due to the limited knowledge of the mechanisms involved in UVM metastasis and progression [3, 4]. Although more and more advancements in the managements of UVM patients have also occurred, the long-term survivals remain poor [5, 6]. Therefore, it is an urgent necessity to identify sensitive diagnostic and prognostic biomarkers to determine optimal treatment modalities for CRC patients.

In recent years, many nonprotein-coding genes, accounting for about 75% of the genome, have been identified due to the tremendous progress of genome and transcriptome sequencing [7, 8]. Long noncoding RNAs (lncRNAs) are RNAs > 200 nucleotides which are transcribed from nonprotein-coding genes [9]. Enhancer RNAs (eRNAs) are a subclass of lncRNAs transcribed within gene enhancers [10, 11]. Although the deficiency of the protein-coding ability of eRNAs limits their biological function in cellular progression, more and more evidences suggest that many eRNAs display a potential effect in genetic and epigenetic regulation [12, 13]. Recent reports indicated that several eRNAs are dysregulated in multiple tumors and may play oncogenic or antioncogenic roles in the oncogenesis and progression of various neoplasms via participating in a series of cellular progression, like tumor growth and distant metastasis [14, 15]. In addition, more and more researches have proved the value of eRNAs used as new biomarkers for diagnosis and outcome of tumor patients [16, 17]. However, a large number of eRNAs remained to be clinically identified.

Recently, many researches have reported the functions of eRNAs in several tumors. For instance, the Enhancer RNA-SMAD7e was highly expressed in bladder tumor, and its silence inhibited the proliferation, migration, and invasion of cancer cells [18]. Li et al. reported that low enhancer RNA SLIT2 expression predicted a poor outcome of breast cancer patients, and its overexpression suppressed bone metastasis of breast cancer via modulating MAPK/c-Fos pathway [19]. However, the expression and function of eRNAs in UVM were rarely reported. In this research, our group aimed to identify survival-related eRNAs in UVM based on TCGA and GEO datasets.

## 2. Materials and Methods

### 2.1. Data Collection

The microarray dataset GSE22138 and the clinical information were downloaded from the GEO databases (https://www.ncbi.nlm.nih.gov/). GSE22138 including 63 tumor samples was performed on the GPL570 platform. Clinical data, sequencing data of RNAs, and survival data for 33 tumor types were collected from TCGA datasets. All expressing data of RNAs were translated into log2 (FPKM+1). GTF annotation files were applied to change Ensemble transcript IDs. The list of eRNA information was obtained online tools. The expressing data of the eRNAs were extracted from TCGA-UVM.

### 2.2. The Identification of Survival-Related eRNAs

Kaplan-Meier assays were applied to analyze the associations between the expressions of eRNAs and survivals by the use of R package. The log-rank tests were applied to examine distinct differences of survival curves stratified by eRNAs. The *p* < 0.05 was considered as indicating a statistically significant difference. The prognostic values of eRNAs were further explored by multivariate assays through the R package survival. Gene Expression Profiling Interactive Analysis (GEPIA) database is an online tool which can be used to analyze expressing and clinical data of tumor patients from TCGA and the GTEx projects [20]. “Survival” modules of GEPIA were applied to examine the associations between expressions of eRNAs and outcome of UVM patients.

### 2.3. Enrichment Analysis of Gene Ontology (GO) and Kyoto Encyclopedia of Genes and Genomes (KEGG) Pathway

clusterProfiler package was applied for GO enrichment assays and KEGG pathway assays for the dysregulated genes [21]. GO enrichment assays mainly described the molecular functions (MFs), cellular components (CCs), and biological processes (BPs), related to the dysregulated genes. KEGG pathway assays indicated tumor-associated pathways related to dysregulated genes. Adjusted *p* value < 0.05 was used as the cutoff standard.

### 2.4. Verification in Pan-Cancer

Survival analysis was conducted to estimate the prognostic values of eRNA expressions on overall survival in pan-cancer. *p* < 0.05 was considered statistically significant. A survival curve was plotted for eRNAs in tumors that met the criteria.

### 2.5. Statistical Analysis

All statistical analyses are based on R language 3.6.1 version (Boston, Massachusetts, USA) and attached packages.

## 3. Results

### 3.1. The Identification of Survival-Related eRNAs in UVM

**Figure 1:**
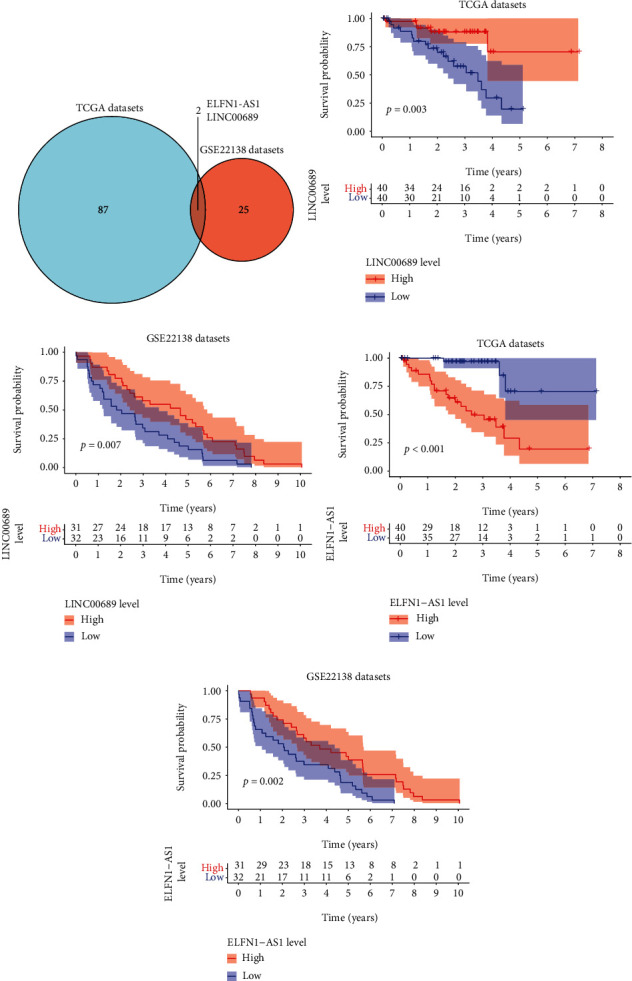
Identification of survival-related eRNAs in UVM. (a) Venn diagram of survival-related eRNAs in TCGA-UVM and GSE22138 datasets. (b, c) Survival curves of OS between LINC00689-high and LINC00689-low patients with UVM based on TCGA and GSE22138. (d, e) Survival curves of OS between ELFN1-AS1-high and ELFN1-AS1-low patients with UVM based on TCGA and GSE22138.

To identify survival-related eRNAs, we analyzed TCGA datasets and found 89 survival-related eRNAs in UVM (Supplementary Table S1). Based on GSE22138 datasets, we found 27 survival-related eRNAs in UVM (Supplementary Table S2). The results of Venn diagram revealed that only two eRNAs (LINC00689 and ELFN1-AS1) were overlapped in both two datasets (Figure 1(a)). Survival assays revealed that patients with low LINC00689 expression showed a shorter OS than those with high LINC00689 expression in both TCGA (*p* = 0.003, Figure 1(b)) and GSE22138 (*p* = 0.007, Figure 1(c)) datasets. Furthermore, we found that high ELFN1-AS1 expression was associated with a shorter OS of UVM patients from TCGA datasets (*p* < 0.001, Figure 1(d)). However, in GSE22138 datasets, a contrary result was observed (Figure 1(e)).

### 3.2. The Potential of LINC00689 and ELFN1-AS1 Used as Novel Prognostic Biomarkers

**Figure 2:**
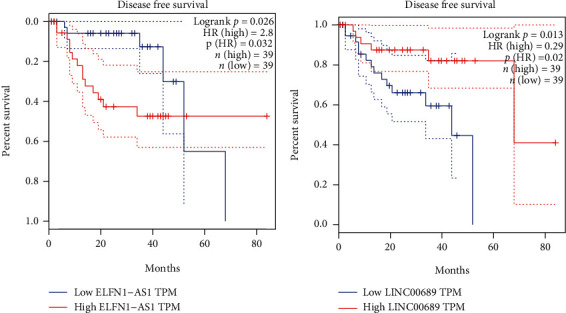
GEPIA was used to study the association between the expressions of (a) ELFN1-AS1 and (b) LINC00689 and disease-free survival of UVM patients based on TCGA datasets.

**Figure 3:**
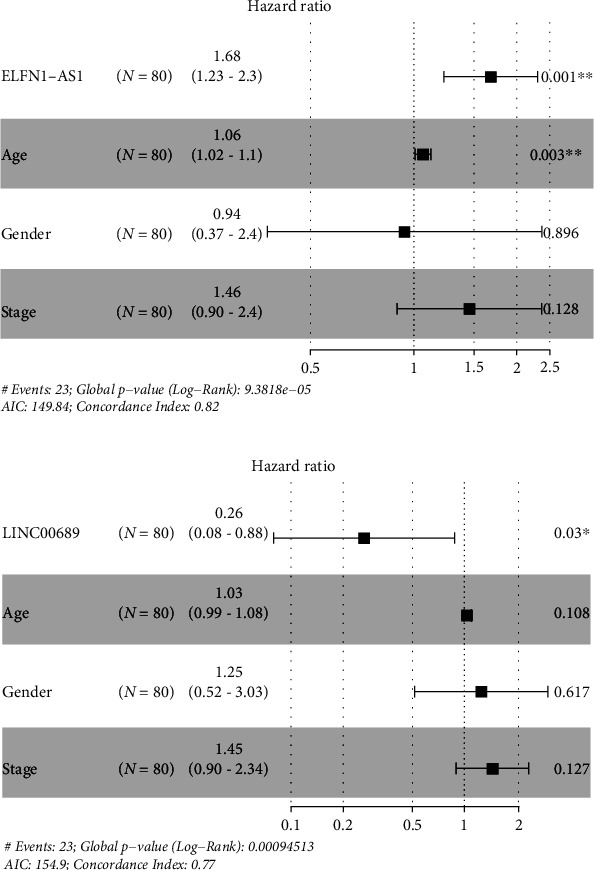
Forest plots of the results of multivariate Cox regression analyses of (a) ELFN1-AS1 and (b) LINC00689. ^∗∗^*p* < 0.01 and ^∗^*p* < 0.05.

Then, we explored GEPIA and found that high ELFN1-AS1 expression predicted a shorter OS (Figure 2(a)), and high LINC00689 expression predicted a longer OS (Figure 2(b)). To further delve into the prognostic values of LINC00689 and ELFN1-AS1 expressions in UVM, we performed multivariate analysis based on TCGA datasets and found that both LINC00689 (HR = 1.68, *p* < 0.001, Figure 3(a)) and ELFN1-AS1 (HR = 0.26, *p* = 0.03, Figure 3(b)) were independent prognostic factors in UVM.

### 3.3. Functional Enrichment Analysis

**Figure 4:**
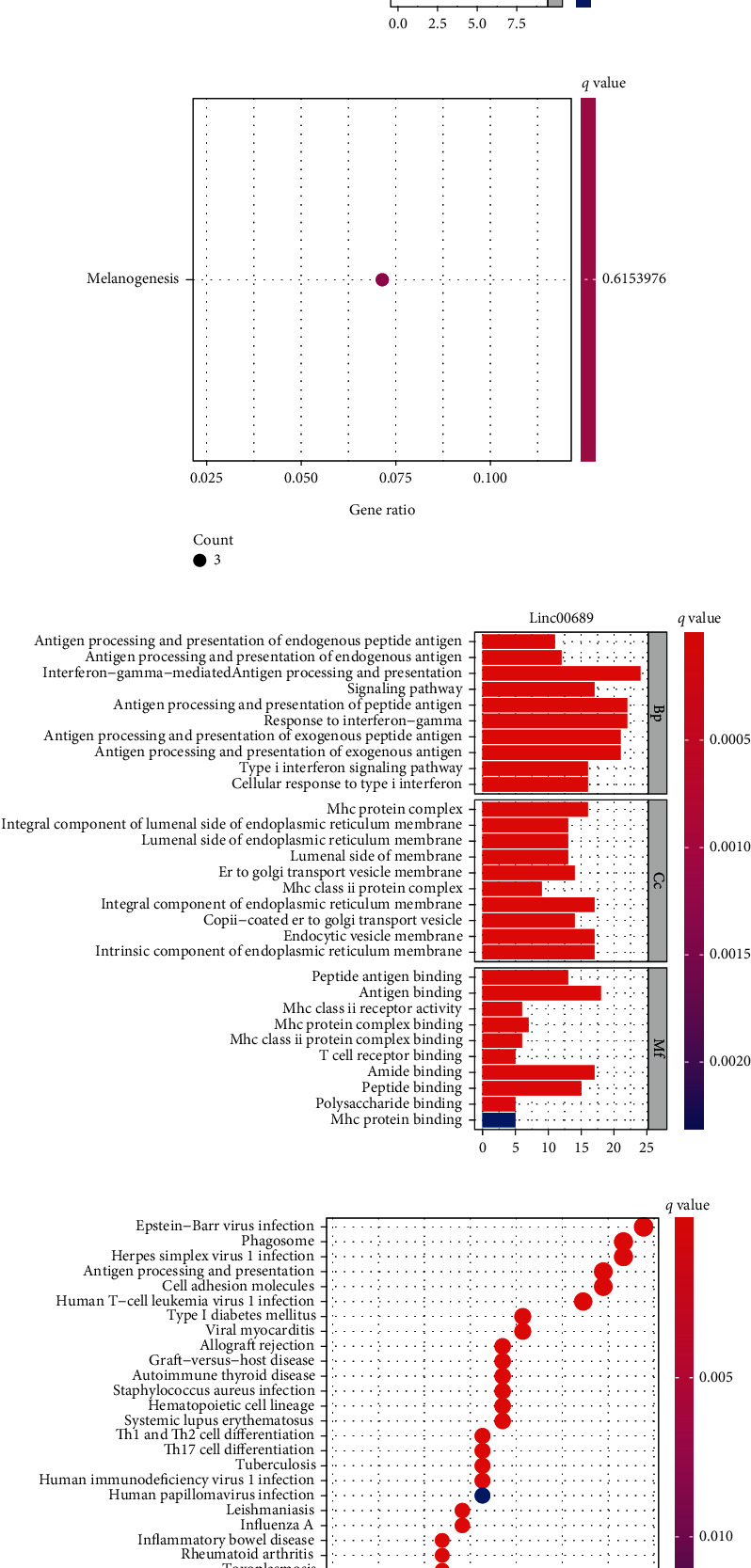
(a) GO and (b) KEGG pathway analysis of differentially expressed genes between high-ELFN1-AS1-expression group and low-ELFN1-AS1-expression group. (c) GO and (d) KEGG pathway analysis of differentially expressed genes between high-LINC00689-expression group and low-ELFN1-AS1-expression group.

To explore the possible functions of LINC00689 and ELFN1-AS1 in UVM, we divided all UVM patients into two groups (high and low) based on the mean expression of LINC00689 and ELFN1-AS1. Then, we screened the dysregulated genes between two groups. Subsequently, we completed GO analysis using the “clusterProfiler” R package for ELFN1-AS1 and found that in the BP group, the dysregulated genes were mainly involved in negative regulation of G protein-coupled receptor signaling pathway. In the CC, the dysregulated genes were mainly involved in collagen-containing extracellular matrix. In the MF group, the dysregulated genes primarily existed in RNA polymerase II activating transcription factor binding (Figure 4(a)). KEGG assays displayed that the dysregulated genes are mainly enriched in melanogenesis (Figure 4(b)). On the other hand, for LINC00689, we observed that in the BP group, the dysregulated genes were mainly involved in antigen processing and presentation of endogenous peptide antigen, antigen processing and presentation of endogenous antigen, and antigen processing and presentation of peptide antigen. In the CC, the dysregulated genes were mainly involved in lumenal side of endoplasmic reticulum membrane, integral component of lumenal side of endoplasmic reticulum membrane, and MHC protein complex. In the MF group, the dysregulated genes primarily existed in peptide antigen binding, antigen binding, and MHC class II receptor activity (Figure 4(c)). KEGG assays displayed that the dysregulated genes are mainly enriched in Epstein-Barr virus infection, phagosome, herpes simplex virus 1 infection, antigen processing and presentation, and cell adhesion molecules (Figure 4(d)).

### 3.4. Pan-Cancer Verification

**Figure 5:**
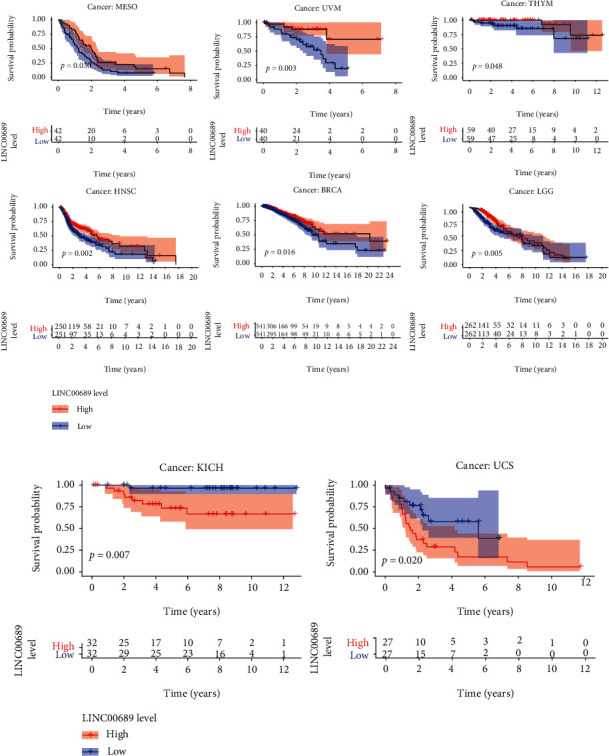
(a, b) Kaplan-Meier assays for LINC00689 in pan-cancer (*p* < 0.05).

**Figure 6:**
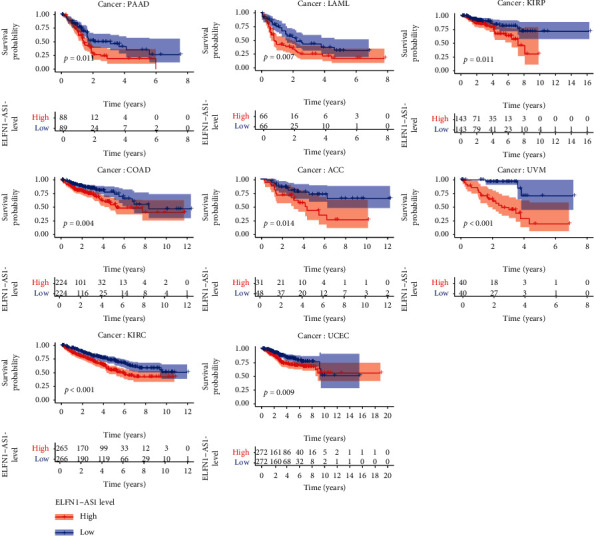
Kaplan-Meier assays for ELFN1-AS1 in pan-cancer (*p* < 0.05).

To examine the prognostic roles of LINC00689 and ELFN1-AS1 in pan-cancer, our group carried out survival assays. We observed that LINC00689 was related to survival in eight tumors, namely, MESO, UVM, THYM, HNSC, BRCA, LGG, KICH, and UCS. The survival curves for LINC00689 in eight tumors are exhibited in Figures 5(a) and 5(b). We found that high LINC00689 expression predicted a longer overall survival in MESO, UVM, THYM, HNSC, BRCA, and LGG (Figure 5(a)), while predicted a shorter overall survival in KICH and UCS (Figure 5(b)). Besides, we observed that ELFN1-AS1 was also associated with survival in eight tumors, namely, PAAD, LAML, KIRP, COAD, ACC, UVM, KIRC, and UCEC (Figure 6).

## 4. Discussion

The progression of metastasis is the main reason for death from UVM [22]. Early diagnosis and an optimized therapeutic schedule based on the prediction of possible prognosis of UVM patients are very important for the reduction of metastatic cases [23, 24]. In recent years, more and more studies reported that eRNAs play a critical role in tumor progression, and their frequent upregulation and stable existence in tumor tissues and blood of patients suggest the potential of eRNAs used as novel biomarkers [23, 25]. Several eRNAs have been confirmed to be positively associated with long-term survival of various tumor patients and have a diagnostic value in distinguishing tumor tissues from normal specimens with high sensitivity and specificity, such as FAM120AOS, LINC00513, and EMX2OS which may be even better than the previously reported biomarkers [26–28]. However, the prognostic values of eRNAs in UVM were rarely reported.

In this study, we identified 87 survival-related eRNAs in UVM by analyzing TCGA datasets and 25 survival-related eRNAs in UVM by analyzing GSE22138. Only two genes including LINC00689 and ELFN1-AS1 overlapped in both two datasets. Previously, the effects of LINC00689 and ELFN1-AS1 in tumor progression have been reported in several tumors. For instance, Zhan and his group reported that LINC00689 expressions were distinctly increased in glioma, and its silence suppressed the proliferation and metastasis of glioma cells via mediation of miR-526b/IGF2BP1 [29]. In colorectal cancer, a significant overexpression of LINC00689 was observed, and its dysregulation was involved in tumor growth, drug resistance, and migration through miRNA-31-5p/YAP in colorectal cancer [30]. On the other hand, ELFN1-AS1 was shown to exhibit an increased level in ovarian cancer, and its overexpression accelerated cell proliferation and migration through the modulation of miRNA-497-3p/CLDN4 [31]. Zhang et al. reported that ELFN1-AS1 predicted a poor prognosis of esophageal cancer patients and promoted the metastasis abilities of tumor cells via increasing GFPT1 through modulating miRNA-183-3p [32]. These findings suggested the above two eRNAs as oncogenes in tumor progression. However, the potential function of LINC00689 and ELFN1-AS1 has not been investigated in UVM.

In this study, we observed that patients with low LINC00689 expressions exhibited a shorter OS and DFS than those with high LINC00689 expressions in both TCGA and GSE22138 datasets. However, based on TCGA datasets, we observed that high ELFN1-AS1 expressions predicted a poor prognosis, while the results of GSE22138 exhibited a contrary result. Thus, more samples are needed to further confirm the possible function of ELFN1-AS1 on clinical outcome of UVM patients. Importantly, multivariate analysis results indicated that both LINC00689 and ELFN1-AS1 were independent prognostic factors in UVM. To explore the possible mechanisms involved in LINC00689 and ELFN1-AS1 effects on UVM progression, we performed KEGG assays and found that LINC00689 could influence the survivals of UVM patients via herpes simplex virus 1 infection, Epstein-Barr virus infection, human T-cell leukemia virus 1 infection, and human papillomavirus infection. However, the results for ELFN1-AS1 are not significant. Furthermore, pan-cancer assays demonstrated that ELFN1-AS1 was related to survivals in eight types of tumors (PADD, LAML, KIRP, COAD, ACC, UVM, KIRC, and UCEC), and LINC00689 was related to survivals in eight types of tumors (MESO, UVM, THYM, HNSC, BRCA, LGG, KICH, and UCS). Our findings suggested LINC00689 and ELFN1-AS1 as important regulators in UVM progression.

Several limitations should be noted in this research. Firstly, the sample size was relatively small, and large clinical trials were necessary to for confirm our findings. Secondly, the expression level of LINC00689 and ELFN1-AS1 in this study was arbitrary. The cutoff level of LINC00689 and ELFN1-AS1 in tumor tissue to predict prognosis remained to be established. Thirdly, the functional mechanisms were not explored in vitro and in vivo in this article. In the future, we will finish the mechanisms and associations with eRNAs related to UVM progression.

## 5. Conclusion

We identified novel UVM-related eRNAs, ELFN1-AS1 and LINC00689 which could be used as a potential marker for UVM patients. More in-depth studies are necessary to further demonstrate the prognostic and diagnostic values of ELFN1-AS1 and LINC00689 in UVM patients.

## Data Availability

The data used to support the study are available from the corresponding author upon request.

## Supplementary Materials

Supplementary 1 Supplementary Table S1: identification of the survival-related eRNAs in UVM based on TCGA datasets.

Supplementary 2 Supplementary Table S2: identification of the survival-related eRNAs in UVM based on GSE22138 datasets.

## References

[1] Jager M. J., Shields C. L., Cebulla C. M. (2020). Uveal melanoma. *Nature Reviews. Disease Primers*.

[2] Chattopadhyay C., Kim D. W., Gombos D. S. (2016). Uveal melanoma: from diagnosis to treatment and the science in between. *Cancer*.

[3] Kaliki S., Shields C. L., Shields J. A. (2015). Uveal melanoma: estimating prognosis. *Indian Journal of Ophthalmology*.

[4] Smit K. N., Jager M. J., de Klein A., Kiliҫ E. (2020). Uveal melanoma: towards a molecular understanding. *Progress in Retinal and Eye Research*.

[5] Rodriguez-Vidal C., Fernandez-Diaz D., Fernandez-Marta B. (2020). Treatment of metastatic uveal melanoma: systematic review. *Cancers*.

[6] Ortega M. A., Fraile-Martínez O., García-Honduvilla N. (2020). Update on uveal melanoma: translational research from biology to clinical practice (review). *International Journal of Oncology*.

[7] Saeidian A. H., Youssefian L., Vahidnezhad H., Uitto J. (2020). Research techniques made simple: whole-transcriptome sequencing by RNA-Seq for diagnosis of monogenic disorders. *The Journal of Investigative Dermatology*.

[8] Wang J., Zhu S., Meng N., He Y., Lu R., Yan G. R. (2019). ncRNA-encoded peptides or proteins and cancer. *Molecular Therapy*.

[9] Kopp F., Mendell J. T. (2018). Functional classification and experimental dissection of long noncoding RNAs. *Cell*.

[10] Ye R., Cao C., Xue Y. (2020). Enhancer RNA: biogenesis, function, and regulation. *Essays in Biochemistry*.

[11] Bose D. A., Berger S. L. (2017). eRNA binding produces tailored CBP activity profiles to regulate gene expression. *RNA Biology*.

[12] Sartorelli V., Lauberth S. M. (2020). Enhancer RNAs are an important regulatory layer of the epigenome. *Nature Structural & Molecular Biology*.

[13] Field A., Adelman K. (2020). Evaluating enhancer function and transcription. *Annual Review of Biochemistry*.

[14] Chen H., Liang H. (2020). A high-resolution map of human enhancer RNA loci characterizes super-enhancer activities in cancer. *Cancer Cell*.

[15] Lee J. H., Xiong F., Li W. (2020). Enhancer RNAs in cancer: regulation, mechanisms and therapeutic potential. *RNA Biology*.

[16] Shigeyasu K., Toden S., Ozawa T. (2020). The PVT1 lncRNA is a novel epigenetic enhancer of MYC, and a promising risk-stratification biomarker in colorectal cancer. *Molecular Cancer*.

[17] Boon R. A., Jaé N., Holdt L., Dimmeler S. (2016). Long noncoding RNAs: from clinical genetics to therapeutic targets?. *Journal of the American College of Cardiology*.

[18] Che W., Ye S., Cai A., Cui X., Sun Y. (2020). CRISPR-Cas13a targeting the Enhancer RNA-SMAD7e inhibits bladder cancer development both in vitro and in vivo. *Frontiers in Molecular Biosciences*.

[19] Li P., Lin Z., Liu Q. (2021). Enhancer RNA SLIT2 inhibits bone metastasis of breast cancer through regulating P38 MAPK/c-Fos signaling pathway. *Frontiers in Oncology*.

[20] Tang Z., Li C., Kang B., Gao G., Li C., Zhang Z. (2017). GEPIA: a web server for cancer and normal gene expression profiling and interactive analyses. *Nucleic Acids Research*.

[21] Yu G., Wang L. G., Han Y., He Q. Y. (2012). clusterProfiler: an R package for comparing biological themes among gene clusters. *Omics: a journal of integrative biology*.

[22] Kaliki S., Shields C. L. (2017). Uveal melanoma: relatively rare but deadly cancer. *Eye (London, England)*.

[23] Hand F., Doherty S., Gullo G., Geoghegan J., Crown J., Hoti E. (2020). Metastatic uveal melanoma: a valid indication for liver resection. *Journal of B.U.ON. Official Journal of The Balkan Union of Oncology*.

[24] Singh A. D., Zabor E. C., Radivoyevitch T. (2021). Estimating cured fractions of uveal melanoma. *JAMA Ophthalmology*.

[25] Li Y., Jia R., Ge S. (2017). Role of epigenetics in uveal melanoma. *International Journal of Biological Sciences*.

[26] Nikas J. B. (2016). Independent validation of a mathematical genomic model for survival of glioma patients. *American Journal of Cancer Research*.

[27] Xue Z., Cui C., Liao Z. (2018). Identification of LncRNA Linc00513 containing lupus-associated genetic variants as a novel regulator of interferon signaling pathway. *Frontiers in Immunology*.

[28] Jiang H., Chen H., Wan P., Song S., Chen N. (2020). Downregulation of enhancer RNA EMX2OS is associated with poor prognosis in kidney renal clear cell carcinoma. *Aging (Albany NY)*.

[29] Zhan W. L., Gao N., Tu G. L., Tang H., Gao L., Xia Y. (2021). LncRNA LINC00689 promotes the tumorigenesis of glioma via mediation of miR-526b-3p/IGF2BP1 axis. *Neuromolecular Medicine*.

[30] Du Y. L., Liang Y., Shi G. Q. (2020). LINC00689 participates in proliferation, chemoresistance and metastasis via miR-31-5p/YAP/*β*-catenin axis in colorectal cancer. *Experimental Cell Research*.

[31] Jie Y., Ye L., Chen H. (2020). ELFN1-AS1 accelerates cell proliferation, invasion and migration via regulating miR-497-3p/CLDN4 axis in ovarian cancer. *Bioengineered*.

[32] Zhang C., Lian H., Xie L., Yin N., Cui Y. (2020). LncRNA ELFN1-AS1 promotes esophageal cancer progression by up-regulating GFPT1 via sponging miR-183-3p. *Biological Chemistry*.

