# Supplementary material for: Comprehensive Analysis of Enhancer RNAs Identifies LINC00689 and ELFN1-AS1 as Novel Prognostic Biomarkers in Uveal Melanoma
Source: Dis Markers. 2022 Feb 23;2022:5994800. doi: 10.1155/2022/5994800 (PMC8892034; doi:10.1155/2022/5994800)
Supplement: Supplementary 1 — Supplementary Table S1: identification of the survival-related eRNAs in UVM based on TCGA datasets. [file 5994800.f1.docx]

**Table S1:** Identification of the survival-related eRNAs in UVM based on TCGA datasets.

| gene | KM | gene | KM |
| --- | --- | --- | --- |
| AC116351.1 | 0.003712 | AC000067.1 | 0.00668 |
| ITFG2-AS1 | 0.001728 | C12orf75 | 9.55E-06 |
| AL772337.3 | 0.004942 | AC098934.1 | 3.16E-05 |
| AP002992.1 | 0.000197 | AL590764.1 | 0.000968 |
| LINC00354 | 0.000844 | LINC00404 | 0.000403 |
| AC027117.2 | 5.16E-05 | AC010273.3 | 1.32E-05 |
| LINC02495 | 0.007623 | DARS-AS1 | 0.008976 |
| GAS1RR | 5.85E-05 | AL451069.1 | 0.000243 |
| TMEM9B-AS1 | 0.008541 | CHST12 | 0.000783 |
| LINC02611 | 0.000613 | LINC01356 | 0.007662 |
| LINC02614 | 0.004658 | TPT1-AS1 | 0.001986 |
| ELFN1-AS1 | 4.17E-05 | BAALC-AS1 | 2.14E-05 |
| MIR5689HG | 8.13E-06 | AL772337.2 | 0.005111 |
| LINC00472 | 0.006363 | TSGA10IP | 3.85E-05 |
| SNHG17 | 0.003292 | AL118511.1 | 0.001733 |
| LINC01725 | 0.000175 | BASP1-AS1 | 0.001987 |
| AL031846.1 | 0.002786 | LINC00963 | 1.14E-06 |
| LINC00513 | 0.000413 | MAP4K3-DT | 6.53E-06 |
| LINC00518 | 9.50E-06 | LINC00881 | 0.004188 |
| LINC00689 | 0.002789 | ELFN1 | 0.000278 |
| LINC01762 | 3.30E-05 | MEF2C-AS1 | 0.000252 |
| HCP5 | 0.001856 | MSN | 0.001124 |
| MIR4435-2HG | 9.58E-05 | HCG20 | 0.00138 |
| SLC44A3-AS1 | 1.50E-06 | STX4 | 0.002815 |
| SATB1-AS1 | 0.000116 | EMG1 | 0.007484 |
| AC096751.2 | 0.001479 | LINC01637 | 0.0003 |
| AP003721.4 | 0.007863 | IGHA2 | 0.002793 |
| AC087672.2 | 0.003793 | LINC00486 | 8.22E-05 |
| AL021392.1 | 4.66E-05 | AL021707.2 | 1.84E-05 |
| LINC01914 | 0.006407 | LINC02446 | 0.000105 |
| FRY | 0.00171 | AC022784.1 | 6.54E-06 |
| AC116535.2 | 0.005172 | AC233976.1 | 0.003889 |
| CT69 | 0.001255 | NR2F1-AS1 | 2.95E-06 |
| AC092490.1 | 0.005248 | AL590652.1 | 0.005069 |
| LINC00665 | 3.73E-05 | TMEM225B | 0.001162 |
| AP001107.5 | 0.000247 | AP003390.1 | 3.33E-07 |
| AC046134.2 | 0.002799 | AC078785.1 | 2.60E-05 |
| CDK6-AS1 | 0.002368 | LINC01270 | 2.29E-06 |
| WDFY3-AS2 | 0.000242 | SOX1-OT | 6.71E-05 |
| AC079209.1 | 0.000296 | STK32A-AS1 | 0.000994 |
| LINC02696 | 4.99E-05 | MAL2 | 0.009754 |
| LINC01252 | 0.001197 | SLC38A3 | 0.000242 |
| MYCNUT | 3.41E-06 | LINC02572 | 8.13E-06 |
| LINC01486 | 0.006033 | CCDC26 | 0.00062 |
| AC027117.1 | 2.17E-05 |  |  |
