# Supplementary material for: Comprehensive Analysis of Enhancer RNAs Identifies LINC00689 and ELFN1-AS1 as Novel Prognostic Biomarkers in Uveal Melanoma
Source: Dis Markers. 2022 Feb 23;2022:5994800. doi: 10.1155/2022/5994800 (PMC8892034; doi:10.1155/2022/5994800)
Supplement: Supplementary 2 — Supplementary Table S2: identification of the survival-related eRNAs in UVM based on GSE22138 datasets. [file 5994800.f2.docx]

**Table S2: Identification of the survival-related eRNAs in UVM based on GSE22138 datasets.**

| gene | KM | gene | KM |
| --- | --- | --- | --- |
| A2MP1 | 0.002472 | LHFPL3-AS2 | 0.000255 |
| AC007389.3 | 0.005821 | LINC00452 | 0.00024 |
| AP001189.4 | 0.043483 | LINC00689 | 0.006969 |
| C1orf61 | 0.005419 | LINC01187 | 0.019766 |
| C20orf203 | 0.020368 | LINC01312 | 0.025649 |
| C5orf66 | 7.49E-05 | LINC01339 | 0.005769 |
| ELFN1-AS1 | 0.002442 | MIATNB | 0.040651 |
| EMX2OS | 0.024019 | PAAF1 | 0.026818 |
| GRAMD1B | 0.012234 | RPS10P7 | 0.032291 |
| GRIP2 | 0.00032 | STK3 | 0.013244 |
| HAGLROS | 0.045711 | TMEM161B-AS1 | 0.011747 |
| HIVEP2 | 0.015267 | UCA1 | 4.87E-07 |
| JPX | 0.040612 | WHAMMP2 | 4.11E-13 |
| ZNF295-AS1 | 0.007164 |  |  |
